# Genome-Wide Identification and Analysis of HAK/KUP/KT Potassium Transporters Gene Family in Wheat (*Triticum aestivum* L.)

**DOI:** 10.3390/ijms19123969

**Published:** 2018-12-10

**Authors:** Xiyong Cheng, Xiaodan Liu, Weiwei Mao, Xurui Zhang, Shulin Chen, Kehui Zhan, Huihui Bi, Haixia Xu

**Affiliations:** Agronomy College of Henan Agricultural University, National Key Laboratory of Wheat and Maize Crop Science, and Collaborative Innovation Center of Henan Grain Crops, Zhengzhou 450046, Henan, China; xyc634@163.com (X.C.); liuxiaodannd1106@163.com (X.L.); 15294621727@163.com (W.M.); zhangxurui95@163.com (X.Z.); cls111cx@163.com (S.C.); kh486@163.com (K.Z.)

**Keywords:** abiotic stress, gene family, HAK/KUP/KT, potassium deficiency, wheat (*Triticum aestivum* L.)

## Abstract

In plants, the HAK (high-affinity K^+^)/KUP (K^+^ uptake)/KT (K^+^ transporter) family represents a large group of potassium transporters that play important roles in plant growth and environmental adaptation. Although *HAK/KUP/KT* genes have been extensively investigated in many plant species, they remain uncharacterized in wheat, especially those involved in the response to environmental stresses. In this study, 56 wheat *HAK/KUP/KT* (hereafter called *TaHAK*s) genes were identified by a genome-wide search using recently released wheat genomic data. Phylogenetic analysis grouped these genes into four clusters (Ι, II, III, IV), containing 22, 19, 7 and 8 genes, respectively. Chromosomal distribution, gene structure, and conserved motif analyses of the 56 *TaHAK* genes were subsequently performed. In silico RNA-seq data analysis revealed that *TaHAK*s from clusters II and III are constitutively expressed in various wheat tissues, while most genes from clusters I and IV have very low expression levels in the examined tissues at different developmental stages. qRT-PCR analysis showed that expression levels of *TaHAK* genes in wheat seedlings were significantly up- or downregulated when seedlings were exposed to K^+^ deficiency, high salinity, or dehydration. Furthermore, we functionally characterized *TaHAK1b-2BL* and showed that it facilitates K^+^ transport in yeast. Collectively, these results provide valuable information for further functional studies of *TaHAKs*, and contribute to a better understanding of the molecular basis of wheat development and stress tolerance.

## 1. Introduction

Potassium is an essential plant macronutrient, which comprises up to 10% of plants dry weight [1]. The decline of potassium amount below 10 g kg^−1^ dry weight can cause severe growth defects in many plants [2]. Potassium is involved in a variety of biochemical processes, including protein synthesis, carbohydrate metabolism, and enzyme activation, as well as in a number of physiological processes, such as stomatal regulation and photosynthesis [3]. In addition, potassium can enhance the tolerance of crop plants to various biotic (e.g., diseases and pests) and abiotic (e.g., salt, drought, and chilling) stresses [3,4,5].

Potassium uptake by plant roots and its translocation inside plants are undertaken by numerous K^+^ channels and transporters [6,7]. K^+^ channels generally function under high external potassium concentrations (>0.5 mM) and are recognized as a low affinity transport system. In contrast, K^+^ transporters, which were initially identified as a high affinity system, are capable of functioning at low external concentrations of K^+^ (<0.2 mM) [8]. However, the dividing line between these two types of systems blurs as the functions of an increasing number of K^+^ channels and transporters were revealed [9,10]. In plants, potassium transporters can be grouped into one of four families: KT (K^+^ transporter)/HAK (high-affinity K^+^)/KUP (K^+^ uptake), Trk (Transport of K^+^)/HKT (high-affinity K^+^ transporters), KEA (K^+^ efflux anti-porter), and CHX (cation/hydrogen exchanger) [11,12]. Early reports on bacterial KUP [13], fungal HAK [14], and Arabidopsis KT [15] transporters used a different set of acronyms, leading to the appearance of composite names HAK/KUP/KT or KT/HAK/KUP or KT/KUP/HAK [16,17,18,19]. These composite names are now widely used to refer to the whole family of K^+^ transporters in plants. The HAK/KUP/KT family, which is the largest potassium transporter family, has been divided into four groups known as clusters I, II, III, and IV [11,20].

Plants contain multiple HAK/KUP/KT transporters; they play diverse roles in K^+^ uptake and transport as well as in regulation of plant growth and development, salt tolerance, and osmotic potential regulation [17]. The availability of K^+^ transport mutants in both Arabidopsis and rice has led to a better understanding of their roles in K^+^ uptake and transport. AtHAK5 and AtAKT1 are the two major contributors to K^+^ uptake in Arabidopsis [21]. Under normal K^+^ conditions, the *AtHAK5* transcripts were detected in roots, but not in shoots. Under conditions of K^+^ deficiency, the *AtHAK5* transcripts were present in both roots and shoots, although in roots the levels of expression were higher [22]. In addition, there is evidence that *AtKUP7* is also involved in K^+^ uptake and may partially mediate xylem K^+^ release [23]. In rice, functions of *AtHAK5* and *AtAKT1* are fulfilled by the rice homologs of these genes, *OsHAK1* and *OsAKT1*. It was found that potassium concentrations in the xylem sap, as well as net potassium export rates in *OsHAK1* mutants, are markedly lower than in wild type control plants, particularly in plants pre-treated with low concentrations of potassium (0.1 mM) [24]. Another K^+^ transporter, *OsHAK5*, partially contributes to high-affinity K^+^ uptake, but at higher K^+^ concentrations than *OsHAK1*. *OsHAK5* is strongly expressed in the xylem parenchyma and phloem of root vascular tissues. Its expression is particularly apparent under potassium-deficient conditions, suggesting that *OsHAK5* may be involved in potassium distribution between roots and shoots [25]. Another family member, *OsHAK21*, has the K^+^ transporter activity, but is not directly involved in K^+^ uptake. *OsHAK21* may play a role in long-distance K^+^ distribution between roots and shoots, because its expression was detected mainly in the root vascular system of stele [26].

It was demonstrated that HAK/KUP/KT transporters protect plants against salt stress. For instance, *OsHAK1* is essential for maintaining potassium-mediated growth of rice plants, and confers salt tolerance across low-to-high potassium concentrations. The *OsHAK*1 knockout mutants have stunted root and shoot growth compared to wild type rice plants. The *OsHAK1* mutants grown in a 0.1 mM K^+^ solution produced 50–55% of the root and 45–50% of the shoot dry weight biomasses of wild type plants. Similarly, when grown in a 1 mM K^+^ solution, those were approximately 70–75% and 60–65%, respectively, of wild types. *OsHAK1* overexpression in rice increased K^+^ uptake as well as the ratio of K^+^/Na^+^ [24]. It is known that an increase of K^+^/Na^+^ ratio in plants leads to the enhancement of salt tolerance. Overexpression of *OsHAK5* increased the K^+^/Na^+^ ratio in shoots and subsequently improved plant salt tolerance, as it was indicated by the better shoot growth of transgenic plants than that of wild type plants. Comparatively, *OsHAK5* knockout mutants decreased K^+^/Na^+^ ratio in shoots, which resulted in increased sensitivity of transgenic plants to salt stress [25]. *OsHAK21* was also suggested to have an essential role in the salt stress response. Expression levels of *OsHAK21* considerably increased under conditions of high salinity; particularly, after 4 h of salt stress the transcription level of *OsHAK21* in roots increased 600-fold. *OsHAK21* mutants accumulated less K^+^ and significantly more Na^+^ in both shoots and roots under salt stress than wild type control plants. They also had a remarkably lower K^+^ net uptake rate and higher Na^+^ uptake rate compared to wild type plants. This highlights the importance of *OsHAK21* in maintaining plant K^+^ homeostasis under conditions of salt stress [26].

Furthermore, HAK/KUP/KT transporters also play a role in drought tolerance. Overexpression of *OsHAK1* in rice enhanced drought tolerance at both vegetative and reproductive stages. *OsHAK1* overexpression seedlings (Ox) had decreased levels of lipid peroxidation, increased proline accumulation, and improved activities of antioxidant enzymes compared to control seedlings. Under drought conditions, *OsHAK1-*Ox plants produced 35% more grain yield than wild type plants [27].

Taken together, the above body of research justify the potential value of HAK/KUP/KT family members in crop breading for improved tolerance to high salinity and drought (occurring in arid and semi-arid areas). Although wheat is one of the most important cereal crops worldwide, information about structures and functions of wheat *HAK/KUP/KT* genes is scarce. In this study, 56 wheat *HAK/KUP/KT* genes were identified by a genome-wide search using recently released wheat genome data. We thoroughly analyzed the phylogeny, presence of conserved motifs in proteins, gene structures, locations of genes on chromosomes, and expression patterns of *HAK/KUP/KT* genes in various wheat tissues and under several environmental stresses. In addition, the *TaHAK1b-2BL* gene was functionally characterized and it was demonstrated that it facilitates K^+^ transport in yeast.

## 2. Results

### 2.1. Identification of the TaHAKs in Wheat

A hidden Markov model (HMM) search for proteins containing the K^+^ transporter domain (Pfam accession no. PF02705) was performed using the latest wheat genome database [28]. A total of 56 *TaHAK*s nucleotide sequences were identified, which encoded 25 family members (Table 1). These 25 family members were named according to their corresponding homologs from rice based on a phylogenetic relationship analysis. Letters a, b, or c were added when more than one wheat family member was clustered together with the product of the same rice gene, e.g., TaHAK1a, TaHAK1b, TaHAK1c. Homoeologs within each of the 25 members were further distinguished by the addition of chromosomal arm symbols to the gene/protein names, such as 1AL, 3BS, or 5DS.

As shown in Table 1, ten *TaHAK*s had three homoeologs distributed across A, B, and D genomes, including *TaHAK1b*, *-5*, *-10*, *-13*, *-16*, *-17a*, *-19a*, *-22*, *-23*, and *-24*. Single gene sequence were found for four family members (*TaHAK1c*, *-11*, *-12*, and *-17b*), while the remaining 11 genes possessed two homoeologous sequences each. The length of the putative TaHAK proteins ranged from 686 (*TaHAK4-7AS*) to 916 (*TaHAK23-5BL*) amino acids. The number of transmembrane segments (TMS) ranged from 10 to 14, with the most common being 11–12 TMS (73.2%). All examined TaHAKs were predicted to be localized to the plasma membrane.

### 2.2. Phylogenetic Analysis of TaHAK Proteins

**Figure 1:**
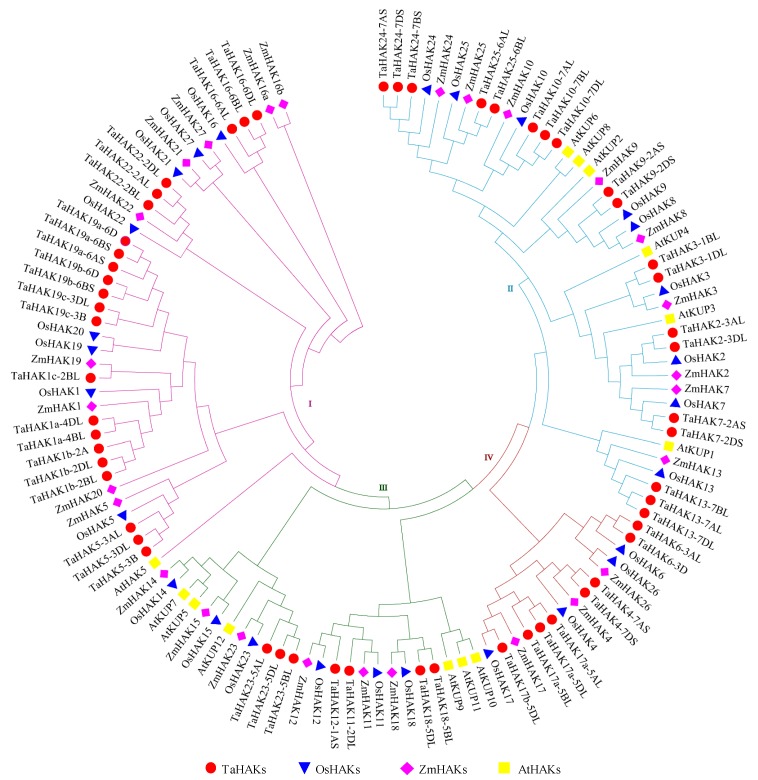
Phylogenetic tree of KUP/HAK/KT family proteins among wheat, rice, maize, and Arabidopsis. The proteins belonging to each of four species are represented by different shapes and colors. The KUP/HAK/KT family proteins were divided into four clusters (I, II, III, IV) and indicated with different colors of lines. The protein loci of rice, maize, and Arabidopsis KUP/HAK/KT family proteins are listed in Table S1. Ta, *Triticum aestivum*; Os, *Oryza sativa*; Zm, *Zea mays*; At, *Arabidopsis thaliana*.

To understand the phylogenetic relationship of the KUP/HAK/KT family proteins, a phylogenetic tree was constructed based on the alignment of the full-length protein sequences of 56 wheat TaHAKs, 27 rice OsHAKs, 27 maize ZmHAKs, and 13 Arabidopsis AtKUP/HAK/KTs (Figure 1). According to the classification criteria used for Arabidopsis and rice [11,22], the wheat HAK proteins were categorized into four clusters. Clusters I, II, III, and IV contained 22, 19, 7, and 8 TaHAK proteins, respectively. Clusters I and II were the most abundant in wheat, maize, and rice, comprising 73%, 67%, and 63% proteins, respectively, while clusters II and III were the most abundant in Arabidopsis, comprising 92% of all AtKUP/HAK/KTs.

### 2.3. Genomic Distribution of TaHAKs

**Figure 2:**
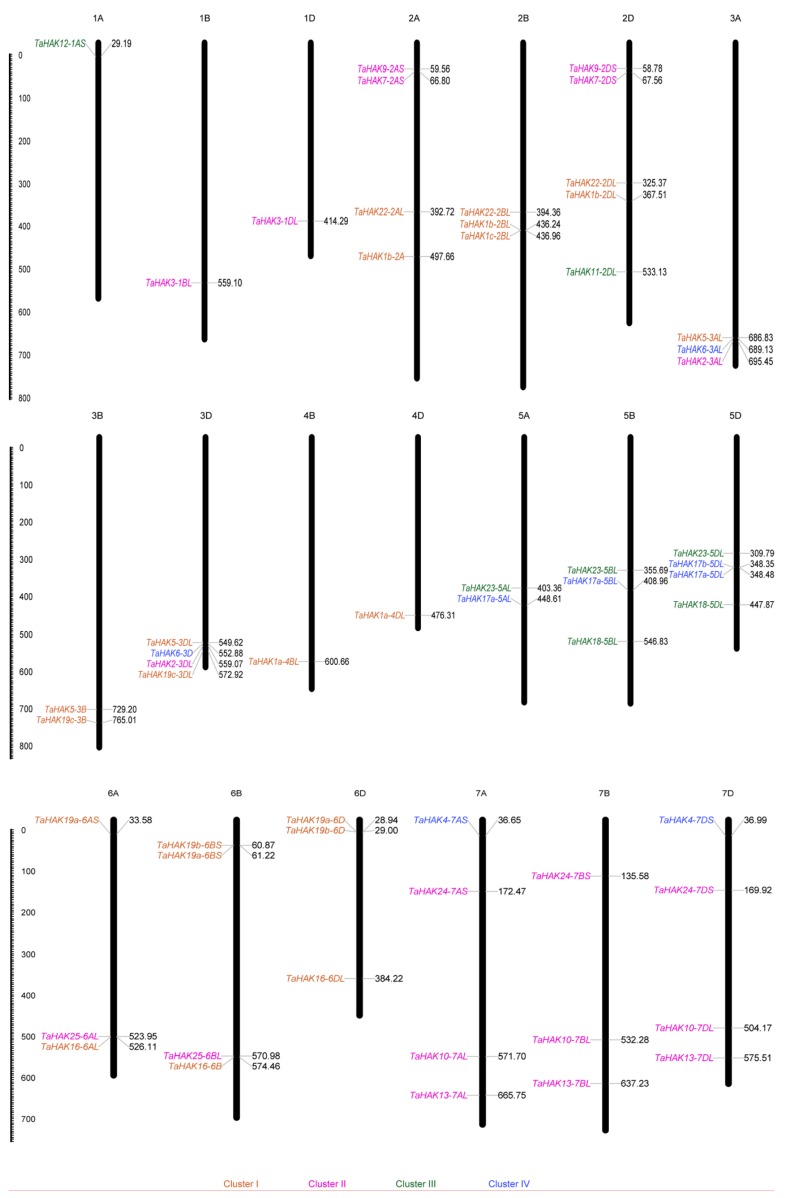
Chromosomal distribution of *TaHAK* genes. The names of chromosomes are shown above each chromosome. The gene names are indicated on the left side and the starting locations are on the right of chromosomes. The *TaHAKs* in each cluster are specified by the same color. The lengths of chromosomes are shown in Mb (Millions of bases).

*TaHAK* genes were mapped to 20 of 21 wheat chromosomes. The physical locations of *TaHAKs* are listed in Table 1. Fifty-six wheat *TaHAK* sequences were approximately evenly distributed among A (17), B (17), and D (22) subgenomes. This was in accordance with the observation that most *TaHAKs* have three homoeologous sequences located on three subgenomes. Nevertheless, *TaHAKs* were non-randomly distributed among different chromosomal groups. The chromosomal groups one and four contained 3 (5.4%) and 2 (3.6%) sequences, respectively. The remaining 51 sequences were more evenly distributed across chromosomal groups two, three, five, six, and seven, ranging from 9 to 12 genes per group (Figure 2).

### 2.4. Gene Structure and Conserved Motif Analyses of TaHAKs

**Figure 3:**
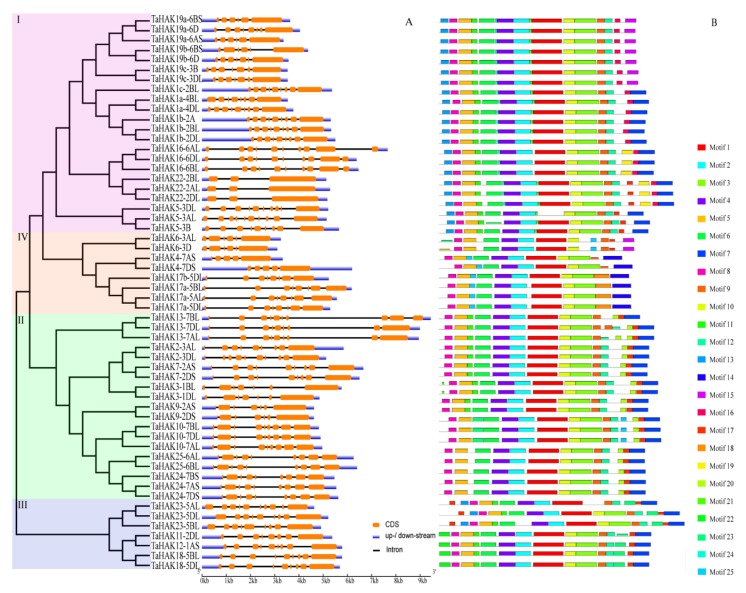
Gene structures and conserved protein motifs of *TaHAK*s. (**A**) Gene structures. *TaHAK* genes are displayed in order based on a phylogenetic analysis of their protein products. Introns, exons and noncoding regions are represented with black lines, orange boxes and blue boxes, respectively. (**B**) Conserved protein motifs. Twenty-five motifs identified in TaHAK proteins marked by different colors.

Based on our phylogenetic analyses, the TaHAK family of proteins was divided into four sub-families (Figure 1 and Figure 3). *TaHAKs* gene structure was revealed using the alignment between CDS and respective genomic sequence by the GSDS [29] online tool (Figure 3A). The examined genes contained 3 to 10 exons and 2 to 9 introns (Figure 3A and Table 1). *TaHAK22* had 3 exons and 2 introns, which was the smallest exon/intron number among all identified *TaHAK* genes. A total of 24 (42.9%) *TaHAK* genes had 9 exons, which was the largest number of exons in examined *TaHAK* genes, followed by 8 (14.3%), 7 (12.5%), 7 (12.5%), 5 (8.9%), 3 (5.4%), and 2 (3.6%) gene sequences, possessing 10, 8, 7, 6, 3, and 5 exons, respectively. Exon/intron numbers also varied inside the same sub-family (cluster I: exon: 3 to 10/intron: 2 to 9, cluster II: exon: 7 to 10/intron 6 to 9, cluster III: exon: 8 to 10/intron: 7 to 9, cluster IV: exon: 5 to 8/intron: 4 to 7), and among the homoeologous genes of the same family member. For example, *TaHAK5-3B* contained 9 exons/8 introns, whereas both *TaHAK5-3AL* and *TaHAK5-3DL* had 10 exons/9 introns. These results are similar to the previously reported results for rice and maize *HAK* genes [11,30].

Protein motifs were analyzed using the online program MEME. In total, 25 conserved motifs were identified in putative TaHAK proteins and designated motifs 1-25 (Figure 3B). More detailed information regarding all conserved motifs is shown in Table S2. As revealed by our NCBI CD (Conserved Domain) search, most conserved motifs were found within the sequence of the K^+^-superfamily domain (Table S3). This result is not surprising, since the 534 amino acid-long “K_trans” domain (PF02705) accounted for a major part of the TaHAK proteins, ranging from 686 to 916 amino acids. Generally, the motifs were almost evenly distributed, and a similar number of motifs was present in TaHAK proteins from each of the four clusters. Motifs 1, 2, 3, 4, 5, 6, 8, 9 and 10 were conserved in all four TaHAK clusters, with a few exceptions where a particular motif was missing in one gene (Figure 3B and Table S4).

We also identified motifs unique to each cluster. They are motifs 13, 16, 21, and 24 (only appeared in cluster I, with few exceptions); motif 20 (only present in cluster II); motif 22 (only found in cluster III); and motifs 14, 18, and 25 (unique to cluster IV, with the exception of motif 25, which was also present in TaHAK10 (cluster II)). In addition, motifs 7 and 12 were present in clusters I, II, and III, but absent in cluster IV. The only exceptions were that motif 7 was missing in TaHAK19s (cluster I) and motif 12 was missing in TaHAK13-7BL (cluster II).

### 2.5. Expression Analysis of TaHAK Genes in Various Wheat Tissues

**Figure 4:**
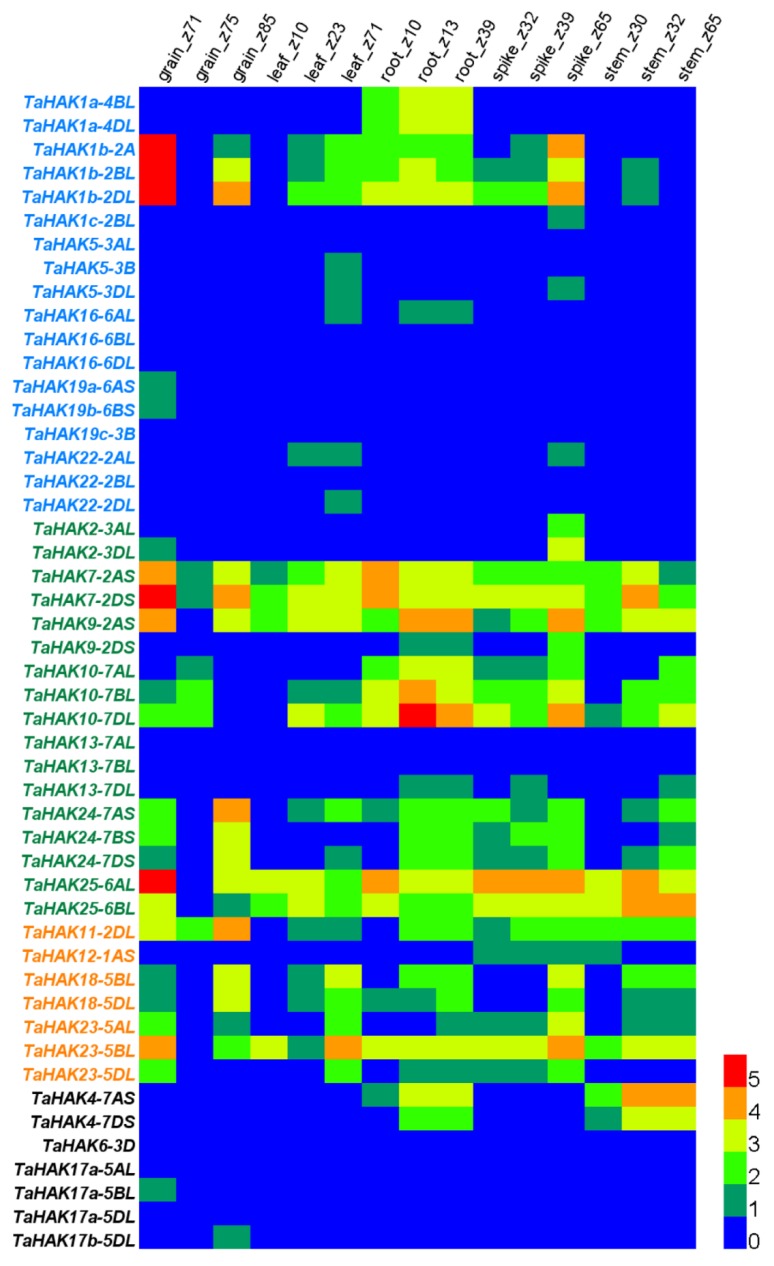
Heat map showing the expression of wheat *TaHAK* genes in various wheat tissues at different developmental stages. RNA-seq data for bread wheat cultivar Chinese spring were obtained from dataset “developmental timecourse in five tissues” presented in WheatExp database [31] Number 0 to 5 represent the range of expression levels (from the lowest to the highest) of the examined genes.

Publicly available RNA-seq databases were used to analyze the expression patterns of *TaHAK* genes in wheat roots, leaves, stems, spikes, and grains. Of the examined 56 genes, data on 49 genes were obtained from RNA-seq databases, while information about the other seven genes (*TaHAK3-1BL* and *-1DL*, *TaHAK6-3AL*, *TaAHK19a-6D* and -*6BS*, *TaHAK19b-6D*, and *TaHAK19c-3DL*) was missing (Figure 4 and Table S5). Most genes in cluster I had low expression levels in almost all tissues, with exception of *TaHAK1a* and *TaHAK1b*. *TaHAK1a-4BL* and *-4DL* were strongly expressed in roots, and *TaHAK1b-2A*, *-2BL,* and *-2DL* were strongly expressed in roots, spikes, and grains, especially in the grain Z71 (2 DAA) stage. For clusters II and III, most genes displayed strong constitutive expression in various tissues, including *TaHAK7*, *TaHAK9-2AS*, *TaHAK10*, *TaHAK24*, *TaHAK25*, *TaHAK11*, *TaHAK18*, and *TaHAK23*. In contrast, *TaHAK2*, *TaHAK13*, and *TaHAK12-1AS* had very low expression levels in most wheat tissues at all examined developmental stages. Most genes from cluster IV had very low expression levels in all tissues, except *TaHAK4-7AS* and *TaHAK4-7DS*, both of which displayed relatively high expression levels in roots and stems.

### 2.6. Expression Analysis of TaHAK Genes under Various Stresses

**Figure 5:**
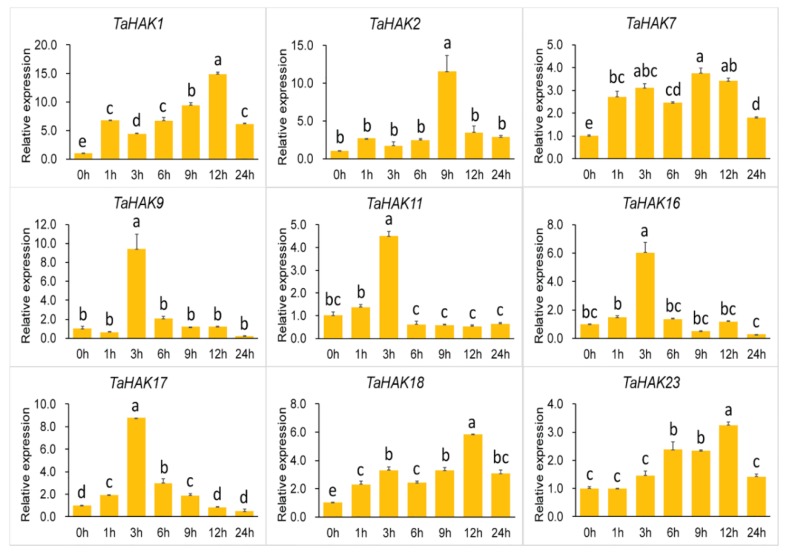
Expression of *TaHAK* genes in response to potassium deficiency stress (0.1 mM K^+^). Expression of *TaHAK* genes was determined by qRT-PCR using total RNA isolated from wheat roots at different time points (0, 1, 3, 6, 9, 12 and 24 h) of the potassium deficiency stress. One-way ANOVA with Duncan’s multiple range test was conducted using SPSS version 20.0. Different letters on top of error bars indicate significant differences at *p* = 0.05 level. Error bars indicate the standard error (SE) of three replicates.

To gain insight into the transcriptional responses of *TaHAK* genes to environmental stresses, the gene expression levels of nine selected *TaHAK* genes were assessed using qRT-PCR after exposure of wheat plants to potassium deficiency, high salinity, and drought. As shown in Figure 5, all nine *TaHAK* genes were upregulated after exposure to potassium deficiency. However, the expression patterns of these genes were different. Basically, expression patterns of all examined genes in plants exposed to the potassium deficiency stress could be divided into two types. The first type is characterized by rapid and continuous upregulation, including *TaHAK1*, *TaHAK7*, *TaHAK18*, and *TaHAK23*. These genes, with the exception of *TaHAK23*, were upregulated after 1 h of exposure to stress. The response of *TaHAK23* to stress was slower, its upregulation became obvious after 6 h of stress. The second type of expression pattern represented the transient upregulation of genes, which included strong upregulation of genes for a short time after exposure to the potassium deficiency stress, followed by a rapid downregulation to the control level, and the low level of gene expression were unchanged for the remaining time of stress. Such type of expression pattern was observed for *TaHAK2*, *TaHAK9*, *TaHAK11*, *TaHAK16*, and *TaHAK17*, most of which showed the highest level of expression after 3 h of stress. The only exception is *TaHAK2*, expression of which reached the maximum after 9 h of exposure to low potassium stress.

**Figure 6:**
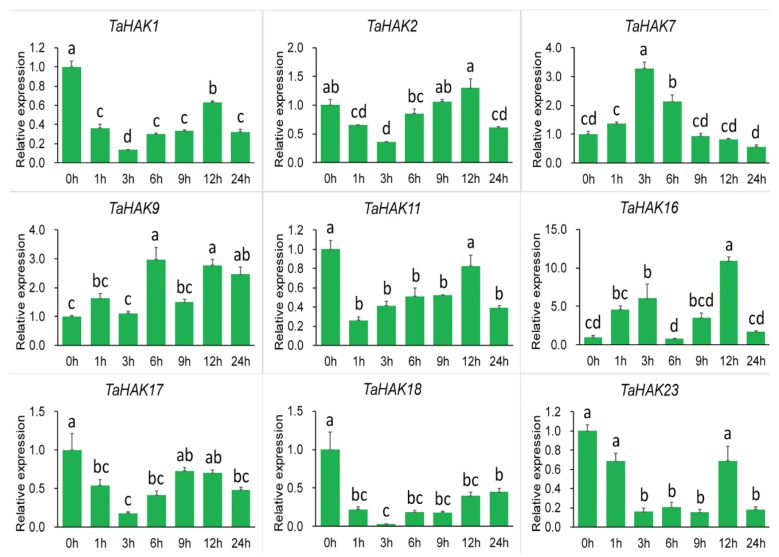
Expression of *TaHAK* genes in response to salt stress (200 mM NaCl). Expression of *TaHAK* genes was determined by qRT-PCR using total RNA isolated from wheat roots at different time points (0, 1, 3, 6, 9, 12 and 24 h) of salt stress. One-way ANOVA with Duncan’s multiple range test was conducted using SPSS version 20.0. Different letters on top of error bars indicate significant differences at *p* = 0.05 level. Error bars indicate the SE of three replicates.

Under salt stress, the expression levels of *TaHAK1*, *TaHAK11*, *TaHAK17*, *TaHAK18*, and *TaHAK23* genes were downregulated. In contrast, under the same stress conditions, expression levels of *TaHAK7*, *TaHAK9*, and *TaHAK16* were markedly increased (Figure 6).

**Figure 7:**
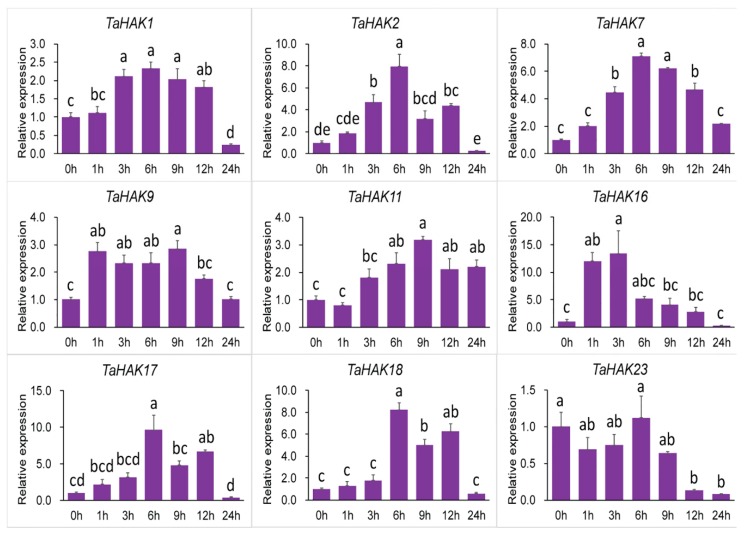
Expression of *TaHAK* genes in response to dehydration simulated by 20% PEG6000. Expression of *TaHAK* genes was determined by qRT-PCR using total RNA isolated from wheat roots at different time points (0, 1, 3, 6, 9, 12 and 24 h) of dehydration. One-way ANOVA with Duncan’s multiple range test was conducted using SPSS version 20.0. Different letters on top of error bars indicate significant differences at *p* = 0.05 level. Error bars indicate the SE of three replicates.

Under dehydration stress simulated by 20% PEG6000, all *TaHAK* genes were upregulated, albeit to different levels (Figure 7). *TaHAK2*, *TaHAK7*, *TaHAK16*, *TaHAK17*, and *TaHAK18* were all significantly upregulated. In particular, *TaHAK16* responded rapidly to dehydration, and its expression reached maximum after 1 h of stress. Four other genes, *TaHAK2*, *TaHAK7*, *TaHAK17*, and *TaHAK18*, reached the maximum expression level after 6 h exposure to dehydration. Comparatively, *TaHAK1*, *TaHAK9*, and *TaHAK11* were moderately upregulated. The expression level of *TaHAK23* remained near the same during the first 9 h of stress and then started to decrease.

### 2.7. TaHAK1b-2BL Functions in Potassium Uptake and Salt Tolerance in Yeast

**Figure 8:**
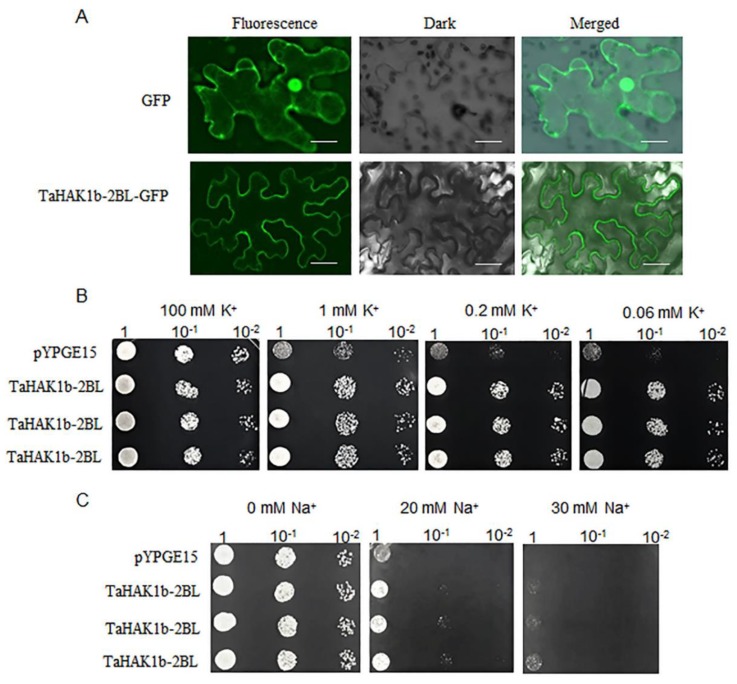
Subcellular location of *TaHAK1b-2BL* and its roles in potassium uptake and salt tolerance. (**A)**. Subcellular location of *TaHAK1b-2BL*. Scale bars = 20 µm. (**B**). Functional complementary assay of *TaHAK1b-2BL* in a high-affinity potassium uptake deficient yeast mutant. Yeasts transformed with the empty vector (pYPGE15) were used as a negative control. The tested potassium concentrations were indicated above each panel. Serial dilutions (10^−1^) of yeast culture were plated. (**C**). Salt tolerance assay of *TaHAK1b-2BL*. The tested sodium concentrations were indicated above each panel. Serial dilutions (10^−1^) of yeast culture were plated.

Most of the reported HAK/KUP/KTs are localized in the plasma membrane [17]. Here, we examined the subcellular location of *TaHAK1b-2BL*, one of *TaHAK1* transporters homologous to *OsHAK1*, which has been previously shown to play a pivotal role in maintaining potassium-mediated growth of rice plants at different salt concentrations. To do this, we generated a construct expressing a fusion protein of TaHAK1b-2BL and green fluorescent protein (GFP). The resulting construct and the control vector (expressing GFP) were introduced into epidermal cells of *Nicotiana benthamiana* leaves. As shown in Figure 8A, the green fluorescence of the GFP control vector was observed in the nucleus, cytosol, and plasma membrane. In contrast, the TaHAK1b-2BL-GFP fusion protein was localized solely in the plasma membrane.

Previous studies revealed that *OsHAK1* could complement the growth defects of a high-affinity potassium uptake deficient yeast mutant. Furthermore, it was shown that the yeast expressing *OsHAK1* could better tolerate high salinity than the yeast harboring an empty vector [24]. In this work, we investigated whether *TaHAK1b-2BL* has a function in potassium uptake using the yeast strain CY162, which is defective in high-affinity potassium uptake. We found that both the yeast transformed with an empty control vector and the yeast transformed with the *TaHAK1b-2BL* construct grew well at 100 mM K^+^ (Figure 8B). However, the yeast expressing *TaHAK1b-2BL* grew better at K^+^ concentrations between 0.06 and 1 mM K^+^ than the control yeast containing an empty vector. In addition, we used the salt-sensitive yeast mutant AXT3K to determine whether the yeast expressing *TaHAK1b-2BL* could tolerate salt stress. As shown in Figure 8C, *TaHAK1b-2BL*-containing yeast and yeast without *TaHAK1b-2BL* demonstrated a similar growth status at 0 mM Na^+^. However, the yeast containing *TaHAK1b-2BL* grew better at 20 mM Na^+^ than the yeast without *TaHAK1b-2BL*. Notably, only the yeast containing *TaHAK1b-2BL* could grow at 30 mM Na^+^.

## 3. Discussion

Thus far, the HAK/KUP/KT family has been reported in a number of plant species, including Arabidopsis [22,32], rice [11,33], maize [30], peach [34], and pear [16]. The gene and protein structures and characteristic features, expression patterns and functions of some *HAK*/*KUP*/*KT* genes have already been characterized. However, a genome-wide analysis of the *HAK* family genes have not yet been performed in wheat (*Triticum aestivum* L.). Recently released wheat genome data allowed us to identify 56 *HAK* genes (including homoeologs) of 25 family members from wheat (Table 1).

In plants, members of the *HAK* gene family display a low level of conservation of their exon/introns structures. The number of exons in wheat *TaHAK* genes ranged from 3 to 10 (Figure 3A and Table 1), which is very similar to that of rice (2 to 10) [11] and maize (3 to 10) [30]. In wheat, 7 out of the 21 *TaHAK* family members with two or three homoeologs contained different exon numbers. For instance, of the three homoeologues of *TaHAK5*, *TaHAK5-3AL*, and *-3DL* had 10 exons each, whereas *TaHAK5-3B* had nine exons. This difference in the exon numbers among homoeologs was also found in other families of wheat genes, such as TaJAZs [35] and MADS-box gene families [36]. This suggests that exon acquisition or loss might have occurred in these gene family during wheat evolution, leading to the various structures of homoeologous genes [35]. It was also found that the exon numbers of orthologous *HAK* genes from different species was rather conserved. For example, *TaHAK22* contained three exons, which was the smallest exon number across all of the *TaHAK* genes. Similarly, *ZmHAK22* and *OsHAK22* genes contained three and two exons, respectively [11,30]. This conservation is in agreement with the fact that homologs in different species normally share similar functions.

The *TaHAK* genes are long and encode for proteins ranging from 686 to 916 amino acids. Of these, 534 amino acids constitute the characteristic “K_trans” domain (PF02705). Although the 25 identified motifs were conserved and evenly present in all TaHAK proteins, several motifs specific to a particular cluster of TaHAKs were found (Figure 3B and Table S4). These specific motifs are likely to contribute to the divergence of wheat TaHAKs. Another characteristic feature of HAK transporters is the presence of the consensus motif GVVYGDLGTSPLY [37]. This motif was a part of the motif 8, one of the 25 conserved protein motifs identified in this study (Table S2).

Spatiotemporal gene expression patterns likely reflect their potential functions. To gain a better insight into the functions of *TaHAK* genes, we performed gene expression analysis for *TaHAK* genes in a variety of wheat tissues using publicly available RNA-seq data. We found that most genes from clusters I and IV had low expression levels across all five tested tissues. In contrast, most genes from clusters II and III were strongly expressed in all tested tissues (Figure 4). These results are in agreement with previous reports on rice and Arabidopsis *HAK* genes. In rice, five genes belonging to clusters II and III—*OsHAK2*, *OsHAK10*, *OsHAK15*, *OsHAK23*, and *OsHAK25*—were expressed in all tissues across all three genotypes [11]. In Arabidopsis, many *AtKT/KUP/HAK*s were expressed in roots, leaves, siliques, and flowers [32]. In fact, 12 out of 13 *AtKT/KUP/HAK* genes belonged to groups II and III. Therefore, the gene expression patterns of *AtKT/KUP/HAKs* were also in accordance with our results that most *TaHAK* genes from clusters II and III are constitutively expressed in all wheat tissues.

The expression of genes from clusters II and III across a variety of tissues explains the observation that cluster II and III KT/KUP/HAKs have a substantial functional diversity [38]. In addition to mediating high- and low-affinity K^+^ uptake, some transporters from clusters II and III are involved in plant growth and development. For instance, *AtKUP4/TRH1* from Arabidopsis has a role in the growth of root hair tips [39] and *AtKT2/KUP2* affects shoot cell expansion [40]. Transporters from clusters II and III may also participate in responses to high salinity, since *AtHAK2*, *AtHAK6*, and *AtHAK11* expression levels have been shown to be altered by salt stress [41]. Notably, *TaHAK1a-4BL* and *-4DL* were strongly expressed in roots, while *TaHAK1b-2A*, *-2BL*, and *-2DL* were strongly expressed in roots, spikes, and grains (Figure 4). Both *TaHAK1a* and *TaHAK1b* were clustered into cluster I together with *OsHAK1*, which was reported to be expressed in whole rice plants, but its strongest expression was observed in roots [20]. Further detailed studies revealed that *OsHAK1* was abundantly expressed in roots, especially at root tips. In contrast, expression levels of this gene in shoots were low, with the exception of meristematic tissues, where expression of *OsHAK1* was rather strong [24]. The expression pattern of *OsHAK1* allows it to play a major role in K^+^ uptake in rice [24].

Transcriptional regulation of K^+^ transporter genes represents a major mechanism in plant responses to low-K^+^ stress [7]. Generally, K^+^ transporter genes are upregulated under conditions of low-K^+^ stress [4]. Previous studies demonstrated that expression of *OsHAK1* and *OsHAK5* genes is induced by potassium deficiency and, consequently, K^+^ uptake in rice roots is enhanced [24,25]. In Arabidopsis, *AtHAK5* is the most prominent gene and the only gene in the HAK/KUP/KT family induced by low-K^+^ stress [32,42]. In this study, we found that all nine tested *TaHAK* genes were upregulated under conditions of potassium deficiency, albeit to different extents (Figure 5). Our results are in good agreement with the previous report on expression of rice *OsHAK* genes. Seventeen *OsHAKs*, with the exception of two undetected genes, were all upregulated by potassium starvation [43]. Interestingly, *TaHAK2*, *TaHAK9*, *TaHAK11*, *TaHAK16*, and *TaHAK17* were upregulated after a short period of potassium deficiency treatment and then rapidly downregulated (transient activation), suggesting that they may be involved in low potassium responses in wheat. *TaHAK1*, *TaHAK7*, *TaHAK18*, and *TaHAK23* exhibited a continuous upregulation, suggesting that they might function in maintaining plants normal growth under potassium deficiency and mediate potassium ion absorption. Under salt stress, the transcripts of three *TaHAK* genes (*TaHAK7*, *TaHAK9* and *TaHAK16*) were clearly increased (Figure 6). Maintaining K^+^ uptake at high Na^+^ is essential for K^+^/Na^+^ homeostasis and salt tolerance [44,45]. Thus, these three genes likely play important roles in mediating K^+^ and Na^+^ homeostasis in wheat under salt stress. Under drought stress, eight of the examined *TaHAK* genes showed increased expression, and only *TaHAK23* was downregulated (Figure 7). Information about expression of *HAK* genes in response to drought is very limited. A previous study revealed that *OsHAK1* expression levels were increased in roots and shoots in response to water deficit. Transgenic rice seedlings overexpressing *OsHAK1* exhibited higher tolerance to drought stress than control plants [27]. Osakabe et al. [46] found that three close homologs, *KUP6, KUP8*, and *KUP2*, may act as key contributors to osmotic adjustment in Arabidopsis by maintaining K^+^ homeostasis during cell growth and under drought.

Chen et al. [24] demonstrated that *OsHAK1* expression could complement the growth defect of the potassium uptake deficient yeast mutant *R5421* under both low (0.05 and 0.1 mM) and high (1mM) external K^+^ conditions. They also found that the yeast transformed with *OsHAK1* could tolerate salt stress much better than control yeast transformed with empty vector in the media containing up to 300 mM NaCl [24]. In this study, we demonstrated that *TaHAK1b-2BL*, a homolog of *OsHAK1*, functions in K^+^ uptake at K^+^ concentrations between 0.06 and 1 mM. Moreover, the salt sensitive yeast mutant *AXT3K* expressing *TaHAK1b-2BL* could tolerate salt stress better than the control yeast at 0 to 30 mM NaCl. *AXT3K* is very sensitive to salt stress due to the dysfunction of its major Na^+^ transporters in the plasma and endosomal membranes [47]. This is likely to be the main reason why the yeast mutant expressing *TaHAK1b-2BL* had stress tolerance only at relatively low (0 to 30 mM) NaCl concentrations. Similarly, Xu et al. showed that the *AXT3K* expressing *TaNHX2* had better salt stress tolerance than control yeast at 0 to 60 mM NaCl [47].

## 4. Materials and Methods

### 4.1. Identification of TaHAK Genes in Wheat

Using the wheat genome data embedded in Ensembl plants database (*Triticum aestivum* TGACv1) [28], we conducted a hidden Markov model (HMM) search using the HMM profile of the K^+^ potassium transporter family (Pfam ID: PF02705) as queries. In total 150 sequences of 81 gene IDs were retrieved. The longest transcript sequence of a gene was retained while the other sequences under the same gene ID were removed. Furthermore, gene sequences without either a start or termination codon as well as incompletely sequenced genes were removed. Remaining sequences were analyzed using the Pfam tool with e-value < e^−5^ [48] and Conserved Domain Database (CDD) provided by the National Center for Biotechnology Information (NCBI) [49], and sequences containing incomplete K^+^ potassium transporter domains were discarded. At the end, 56 non-redundant sequences were identified as wheat *TaHAK* genes. Subcellular localization of each TaHAK protein was predicted using WOLF PSORT software [50] and the transmembrane structure was obtained using TMHMM Server 2.0 online tool [51]. Default parameters were used for all the programs unless otherwise stated.

### 4.2. Phylogenetic Analysis of TaHAKs

The protein sequences for AtKUP/HAK/KTs, OsHAKs, and ZmHAKs were retrieved from the NCBI [52], and these protein loci are listed in Table S1. The full-length proteins of AtHAK/KUP/KTs, OsHAKs, ZmHAKs, and the newly identified TaHAKs were aligned using ClustalW (embedded in MEGA7), and the phylogenetic tree was constructed based on the alignment using MEGA7 [53] with the neighbor-joining method and bootstrap replicates set to 1000.

### 4.3. Physical Localization of TaHAK Genes

To obtain *TaHAKs* chromosomal locations, the CDS of each gene was used to blast the URGI wheat genome database [54] with the expect threshold <e^−10^. The information regarding chromosomal length was obtained from the IWGSC CS RefSeq v.1.0 database [55]. The MapInspect software [56] was used to visualize *TaHAK* genes chromosomal distributions according to their gene starting positions and chromosomal lengths.

### 4.4. Analysis of Gene Structures and Protein Motifs 

The coding and genome sequences of each *TaHAK* gene were downloaded from the wheat database in Ensembl Plants [28]. The *TaHAKs* gene structures were generated using the GSDS online tool [57], and the gene structures were arranged according to the protein positions in the phylogenetic tree constructed by the method of 4.2. The conserved motifs of the deduced TaHAK proteins were identified in the MEME website [58] using the following parameters: the finding motif number was set to 25 and the range of the motif length was set to 5–200 aa.

### 4.5. Plant Materials, Growth Conditions, and Experimental Treatments

Wheat (*Triticum aestivum* L. cv. Aikang58) seeds were grown for five days in culture dishes containing water in a greenhouse at 22 °C with a photoperiod of 16 h light/8 h dark. The five-day-old seedlings were transplanted into a nutrient solution (Table S6), which was replaced every three days until the seedlings reached the three-leaf stage. Roots at the three-leaf stage of seedling development were then subjected to K^+^ deficiency (0.1 mM K^+^) treatment, salt stress (200 mM NaCl), and dehydration (20% PEG6000). Roots were collected at 0, 1, 3, 6, 9, 12 and 24 h after the application of stress and treatments. All plant samples were collected in three biological replicates and were immediately frozen in liquid nitrogen. All samples were then stored at −80 °C until RNA isolation.

### 4.6. RNA Isolation and qRT-PCR

Total RNA was extracted using TransZOL (TransGEN Biotech, Beijing, China) according to the manufacturer’s instructions. cDNA was synthesized using PrimeScript™ RT reagent Kit with gDNA Eraser (Takara, Dalian, China). GoTaq® qPCR Master Mix (Promega, Beijing, China) was used for qPCR amplification in Quantstudio™ 5 (Thermo Fisher, Shanghai, China). The qRT-PCR conditions were as follows: 95 °C for 5 min, followed by 40 cycles of 95 °C for 15 s, and 61 °C for 1 min, and a final extension at 72 °C for 5 min. The wheat β-actin gene was used as an internal reference gene for all qRT-PCR and expression levels were evaluated using the 2^−∆∆*Ct*^ method. Publicly available RNA-seq data of the bread wheat cultivar Chinese Spring was retrieved from the WheatExp [31] website and used to evaluate the expression of wheat HAK genes in different wheat tissues. All qRT-PCR primers are listed in Table S7.

### 4.7. Subcellular Location of the TaHAK1b-2BL Protein

The *TaHAK1b-2BL* ORF sequence was amplified and inserted in frame to upstream *GFP* gene sequence in the 35S-GFP vector using ClonExpress II One Step Cloning Kit (Vazyme, Nanjing, China). All primers are listed in Table S7. The resulting construct was transformed into *Agrobaeterium tumefaeiens* strain GV3101, which was then introduced into leaf epidermal cells of *Nicotiana benthamiana* by agro-infiltration. The transformed plants were allowed to grow for two days at 22 °C with a photoperiod of 16/8 h light/dark before visualization under a confocal laser-scanning microscope (LSM 710, ZEISS, Oberkochen, Germany). 

### 4.8. Functional Complementation Assay of TaHAK1b-2BL in Yeast

The *TaHAK1b-2BL* coding sequence was amplified using the primers listed in Table S7 and cloned into the pYPGE15 vector, which was linearized by double digestion. The vectors pYPGE15-TaHAK1b-2BL and pYPGE15 were then transformed into the potassium uptake-deficient yeast strain CY162 (*MATα*, *ura3-52*, *his3Δ200*, *his4-15*, *trk1Δ*, *trk2Δ1*::pCK64) [59] and the salt-sensitive yeast mutant AXT3K (*Δena1::HIS3::ena4*, *Δnha1::LEU2*, *Δnhx1::KanMX4*) [60], respectively. The yeast transformants were grown to saturation in YNB medium as previously described [61]. These initial yeast cultures were used for making series of dilutions (10^−1^) and 5 μL samples of each diluted culture were plated onto AP plates containing 100, 1, 0.2, and 0.06 mM KCl or 20 and 30 mM NaCl, as indicated. The plates were incubated at 30 °C for 5 d before pictures were taken.

## 5. Conclusions

In this study, we identified 56 wheat *TaHAK* genes from a genome-wide survey of the recently available wheat genome data. Phylogenetic analysis of gene products resulted in identification of four clusters, containing 22, 19, 7, and 8 proteins, respectively. Protein sequences analysis revealed motifs that are conserved in the proteins originating from all four clusters as well as motifs unique for proteins from particular clusters of TaHAKs. Gene expression analysis using publicly available RNA-seq data revealed that *TaHAK*s from clusters II and III are constitutively expressed in various wheat tissues, while most genes in clusters I and IV have very low expression levels in all examined tissues at different developmental stages. The qRT-PCR analysis showed that *TaHAK* genes are differentially up- or downregulated in seedlings subjected to K^+^ deficiency, salinity, or dehydration. Furthermore, functional characterization of the *TaHAK1b-2BL* gene demonstrated that it facilitates K^+^ transport in yeast. Overall, our study provides a systematic description of HAK/KUP/KT potassium transporter genes in wheat. The obtained results deliver valuable information for future functional characterization of *TaHAK*s using transgenic plants, which we are currently planning in our laboratory.

## Figures and Tables

**Table 1:** *TaHAK* genes identified in wheat. Twenty-five TaHAK family members are indicated by white or light grey blocks. TMS, transmembrane segments; S.L., subcellular location; PM, plasma membrane.

Gene Name	Ensembl ID	Amino Acid Length	TMS	Intron No.	Exon No.	S.L.	Gene Location (bp)	
							Start	End
*TaHAK1a-4BL*	TRIAE_CS42_4BL_TGACv1_321685_AA1064100.1	784	11	7	8	PM	600657723	600654569
*TaHAK1a-4DL*	TRIAE_CS42_4DL_TGACv1_343636_AA1137540.1	778	11	8	9	PM	476306655	476302887
*TaHAK1b-2BL*	TRIAE_CS42_2BL_TGACv1_129472_AA0385230.2	768	11	8	9	PM	436243088	436237762
*TaHAK1b-2DL*	TRIAE_CS42_2DL_TGACv1_158352_AA0516430.1	776	11	8	9	PM	367507729	367502225
*TaHAK1b-2A*	TRIAE_CS42_U_TGACv1_641460_AA2095620.1	772	11	8	9	PM	497657505	497652193
*TaHAK1c-2BL*	TRIAE_CS42_2BL_TGACv1_129437_AA0383630.1	774	10	8	9	PM	436956667	436962029
*TaHAK2-3AL*	TRIAE_CS42_3AL_TGACv1_194420_AA0632700.2	784	13	8	9	PM	695496006	695500670
*TaHAK2-3DL*	TRIAE_CS42_3DL_TGACv1_250243_AA0864920.1	788	13	9	10	PM	559072825	559076602
*TaHAK3-1BL*	TRIAE_CS42_1BL_TGACv1_033126_AA0137190.1	818	11	6	7	PM	559097952	559092195
*TaHAK3-1DL*	TRIAE_CS42_1DL_TGACv1_062044_AA0207990.1	817	11	6	7	PM	414287464	414282613
*TaHAK4-7AS*	TRIAE_CS42_7AS_TGACv1_570480_AA1836390.3	686	11	4	5	PM	36648278	36651604
*TaHAK4-7DS*	TRIAE_CS42_7DS_TGACv1_622074_AA2032320.3	725	11	4	5	PM	36992201	36998396
*TaHAK5-3AL*	TRIAE_CS42_3AL_TGACv1_193587_AA0613950.2	788	11	9	10	PM	686834648	686837566
*TaHAK5-3B*	TRIAE_CS42_3B_TGACv1_222217_AA0760010.1	782	12	8	9	PM	729204607	729207978
*TaHAK5-3DL*	TRIAE_CS42_3DL_TGACv1_249200_AA0841080.1	764	11	9	10	PM	549623301	549628488
*TaHAK6-3AL*	TRIAE_CS42_3AL_TGACv1_195362_AA0648570.1	732	11	5	6	PM	689132246	689135499
*TaHAK6-3D*	TRIAE_CS42_U_TGACv1_646122_AA2146080.1	732	10	5	6	PM	552878679	552881787
*TaHAK7-2AS*	TRIAE_CS42_2AS_TGACv1_114941_AA0370050.1	778	13	8	9	PM	66798807	66805468
*TaHAK7-2DS*	TRIAE_CS42_2DS_TGACv1_179048_AA0603870.1	778	13	8	9	PM	67562036	67555857
*TaHAK9-2AS*	TRIAE_CS42_2AS_TGACv1_112740_AA0344400.1	782	11	6	7	PM	59558656	59554035
*TaHAK9-2DS*	TRIAE_CS42_2DS_TGACv1_177265_AA0571140.1	780	11	6	7	PM	58776915	58781534
*TaHAK10-7AL*	TRIAE_CS42_7AL_TGACv1_556774_AA1770580.1	829	11	8	9	PM	571701040	571706004
*TaHAK10-7BL*	TRIAE_CS42_7BL_TGACv1_578444_AA1895080.2	825	11	8	9	PM	532275080	532279899
*TaHAK10-7DL*	TRIAE_CS42_7DL_TGACv1_604392_AA1997720.1	827	11	8	9	PM	504174129	504179023
*TaHAK11-2DL*	TRIAE_CS42_2DL_TGACv1_159022_AA0530870.1	792	14	7	8	PM	533132266	533137640
*TaHAK12-1AS*	TRIAE_CS42_1AS_TGACv1_020203_AA0075600.3	790	14	8	9	PM	29186381	29192163
*TaHAK13-7AL*	TRIAE_CS42_7AL_TGACv1_557049_AA1775770.1	803	11	8	9	PM	665751743	665743564
*TaHAK13-7BL*	TRIAE_CS42_7BL_TGACv1_577019_AA1862890.1	750	11	9	10	PM	637233552	637228550
*TaHAK13-7DL*	TRIAE_CS42_7DL_TGACv1_603280_AA1979940.1	803	11	8	9	PM	575510919	575502903
*TaHAK16-6AL*	TRIAE_CS42_6AL_TGACv1_471561_AA1510890.1	806	11	9	10	PM	526114436	526120099
*TaHAK16-6BL*	TRIAE_CS42_6BL_TGACv1_502060_AA1622780.1	802	11	9	10	PM	574459052	574465508
*TaHAK16-6DL*	TRIAE_CS42_6DL_TGACv1_527466_AA1704460.1	805	11	9	10	PM	384217110	384223492
*TaHAK17a-5AL*	TRIAE_CS42_5AL_TGACv1_374155_AA1191940.2	719	12	7	8	PM	448614349	448619914
*TaHAK17a-5BL*	TRIAE_CS42_5BL_TGACv1_405525_AA1329450.1	719	12	7	8	PM	408955185	408959389
*TaHAK17a-5DL*	TRIAE_CS42_5DL_TGACv1_433406_AA1412260.1	719	12	7	8	PM	348479742	348483109
*TaHAK17b-5DL*	TRIAE_CS42_5DL_TGACv1_434380_AA1434940.1	712	11	7	8	PM	348346780	348352011
*TaHAK18-5BL*	TRIAE_CS42_5BL_TGACv1_407556_AA1357920.1	785	14	8	9	PM	546832103	546826324
*TaHAK18-5DL*	TRIAE_CS42_5DL_TGACv1_433145_AA1403590.1	785	14	8	9	PM	447886169	447880484
*TaHAK19a-6AS*	TRIAE_CS42_6AS_TGACv1_488672_AA1576040.1	735	11	5	6	PM	33582679	33586043
*TaHAK19a-6BS*	TRIAE_CS42_6BS_TGACv1_514904_AA1665660.1	737	11	5	6	PM	61222797	61226427
*TaHAK19a-6D*	TRIAE_CS42_U_TGACv1_644308_AA2138720.1	734	11	7	8	PM	28943744	28939705
*TaHAK19b-6BS*	TRIAE_CS42_6BS_TGACv1_513620_AA1645910.1	736	11	5	6	PM	60865245	60860866
*TaHAK19b-6D*	TRIAE_CS42_U_TGACv1_644372_AA2139050.1	738	11	6	7	PM	28998084	29001654
*TaHAK19c-3B*	TRIAE_CS42_3B_TGACv1_221313_AA0736890.1	745	10	6	7	PM	765005247	765001714
*TaHAK19c-3DL*	TRIAE_CS42_3DL_TGACv1_251389_AA0881060.1	744	10	6	7	PM	574916450	574919986
*TaHAK22-2AL*	TRIAE_CS42_2AL_TGACv1_096842_AA0321670.1	875	12	2	3	PM	392724188	392729472
*TaHAK22-2BL*	TRIAE_CS42_2BL_TGACv1_133206_AA0441840.1	873	12	2	3	PM	394364701	394359570
*TaHAK22-2DL*	TRIAE_CS42_2DL_TGACv1_158627_AA0523560.1	878	12	2	3	PM	325365084	325359910
*TaHAK23-5AL*	TRIAE_CS42_5AL_TGACv1_377379_AA1246300.1	814	10	9	10	PM	403355442	403350814
*TaHAK23-5BL*	TRIAE_CS42_5BL_TGACv1_406332_AA1344100.3	916	12	8	9	PM	355690579	355685671
*TaHAK23-5DL*	TRIAE_CS42_5DL_TGACv1_435669_AA1453280.1	898	12	8	9	PM	309789789	309784581
*TaHAK24-7AS*	TRIAE_CS42_7AS_TGACv1_569122_AA1807860.1	772	11	8	9	PM	172470095	172464552
*TaHAK24-7BS*	TRIAE_CS42_7BS_TGACv1_593324_AA1950450.2	771	13	8	9	PM	135583478	135578011
*TaHAK24-7DS*	TRIAE_CS42_7DS_TGACv1_621622_AA2021390.1	771	11	8	9	PM	169916315	169910689
*TaHAK25-6AL*	TRIAE_CS42_6AL_TGACv1_471048_AA1501690.1	769	12	8	9	PM	523950482	523944232
*TaHAK25-6BL*	TRIAE_CS42_6BL_TGACv1_501501_AA1618220.2	768	13	8	9	PM	570980883	570976570

## Supplementary Materials

Supplementary materials can be found at http://www.mdpi.com/1422-0067/19/12/3969/s1.
