# Supplementary material for: Genome-Wide Identification and Analysis of HAK/KUP/KT Potassium Transporters Gene Family in Wheat (Triticum aestivum L.)
Source: Int J Mol Sci. 2018 Dec 10;19(12):3969. doi: 10.3390/ijms19123969 (PMC6321448; doi:10.3390/ijms19123969)
Supplement: Supplementary file 1 [file ijms-19-03969-s001.zip › Supplementary files/Table S1.docx]

Table S1: Conserved motifs identified from the *TaHAK* genes in wheat*.*

| Motif1 | 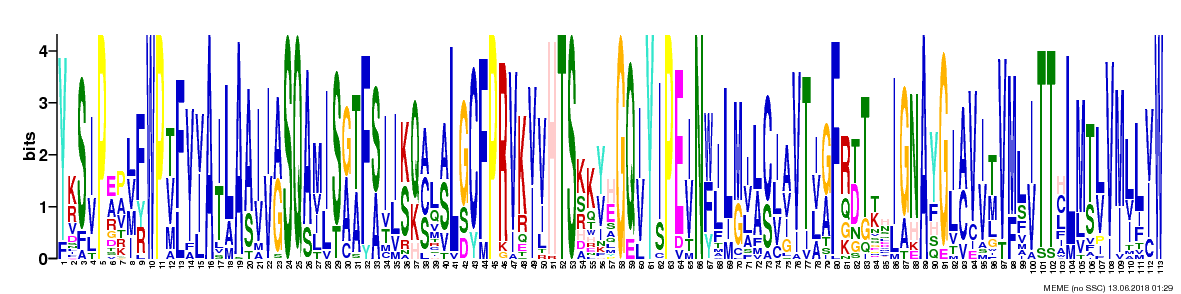   \|  \| \| --- \| |
| --- | --- | --- |
|  | YKSIPEPLFWPTFVVAILAAIIASQAMISGTFSIIKQALALGCFPRVKVVHTSKKYHGQIYIPEINWJLMILCIAVTIGFRTTTHIGNAYGJAVITVMLI |
| Motif2 | 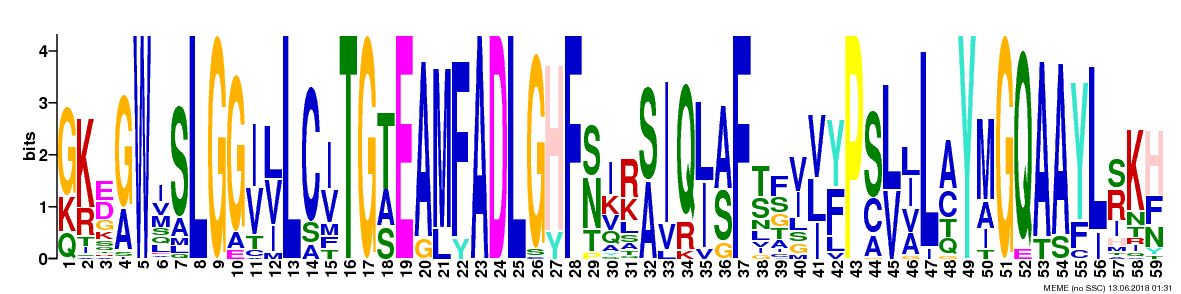 |
|  | GKEGWISLGGILLCITGTEAMFADLGHFSIRSIQJAFTFVVYPSLILAYMGQAAYLSKH |
| Motif3 | 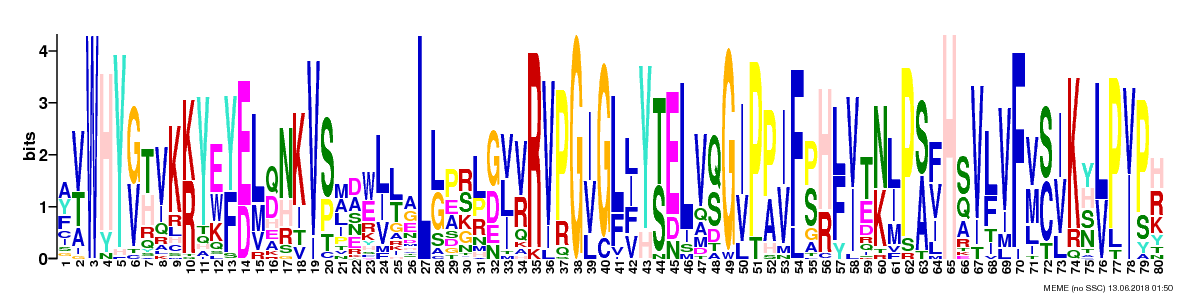 |
|  | AVWHYGTVKKYEYELQNKVSMDWLLALLPRLGVVRVPGIGLLYTELVQGIPPIFPHLVTNJPAFHSVLVFVSIKYLPVPH |
| Motif4 | 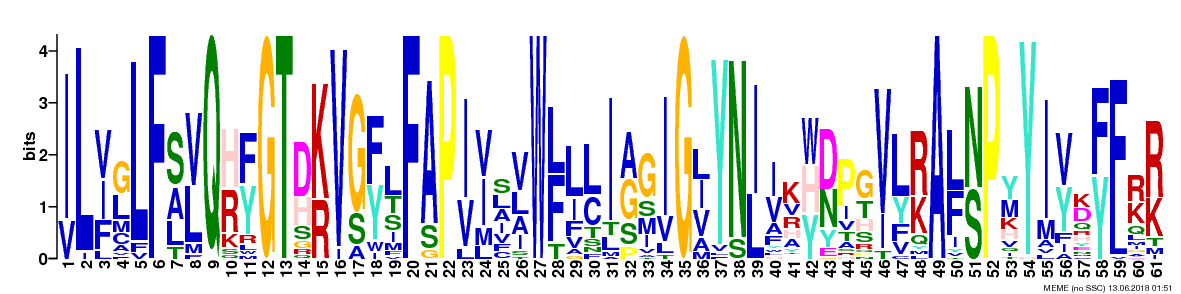 |
|  | ILVGLFSVQHFGTDKVGFLFAPIVSVWLLLIAGIGLYNJIKHBPGVLRALNPYYIVKFFRR |
| Motif5 | 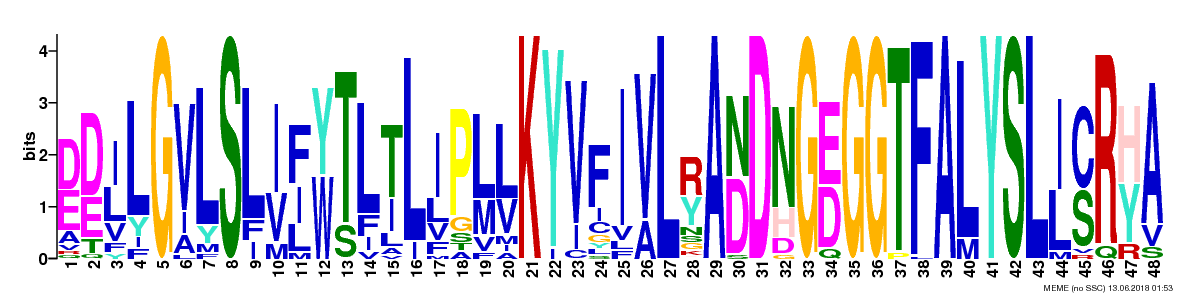 |
|  | DDJLGVLSLIFYTLTLIPLLKYVFIVLRABDNGEGGTFALYSLJCRHA |
| Motif6 | 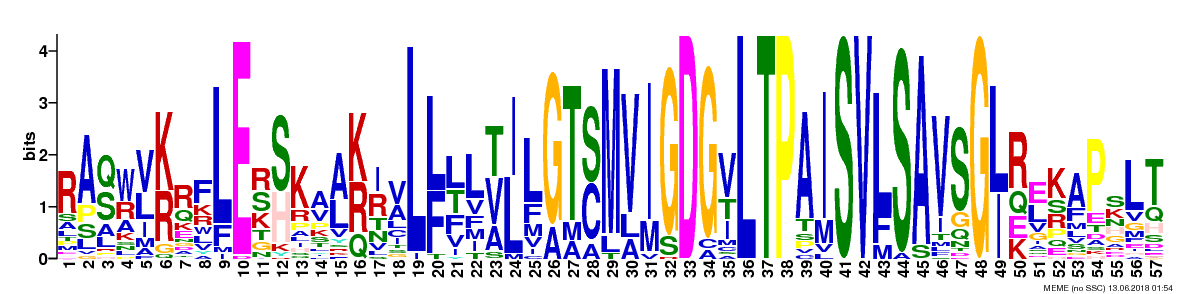 |
|  | RAQWVKRFLERSKAAKIVLLLLTJLGTSMVIGDGVLTPAISVLSAVSGJREKAPSLT |
| Motif7 | 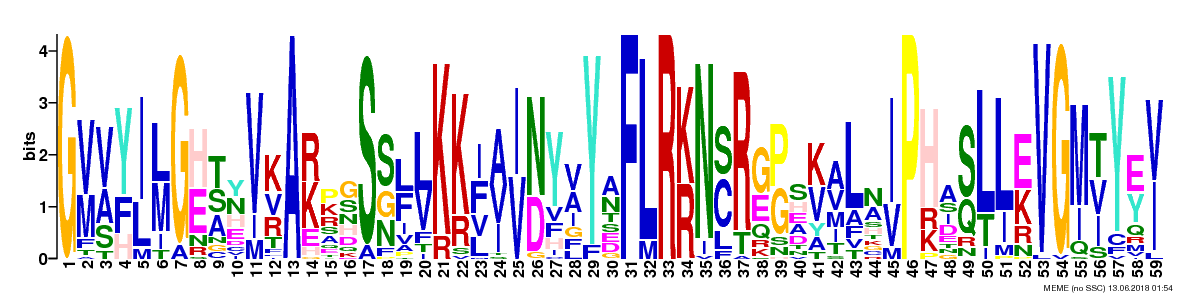 |
|  | GVVYILGHTNVKARPGSSLLKKIAIBYVYAFLRKNSRGPSKALNIPHASLLEVGMTYEV |
| Motif8 | 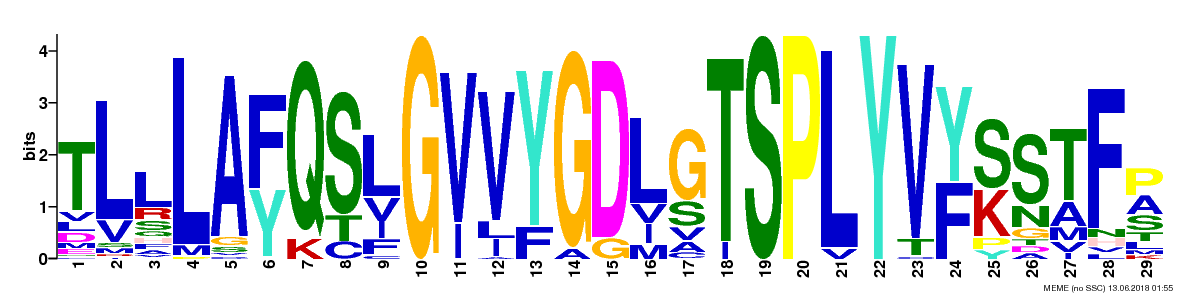 |
|  | TLLLAFQSLGVVYGDLGTSPLYVYSSTFP |
| Motif9 | 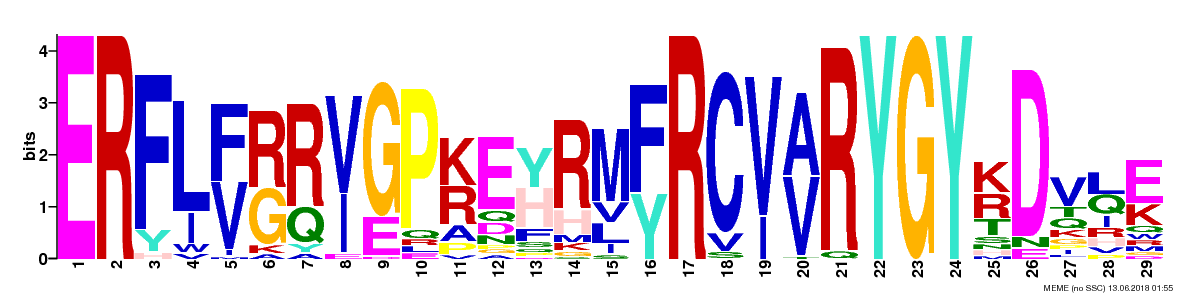 |
|  | ERFLFRRVGPKEYRMFRCVARYGYKDVLE |
| Motif10 | 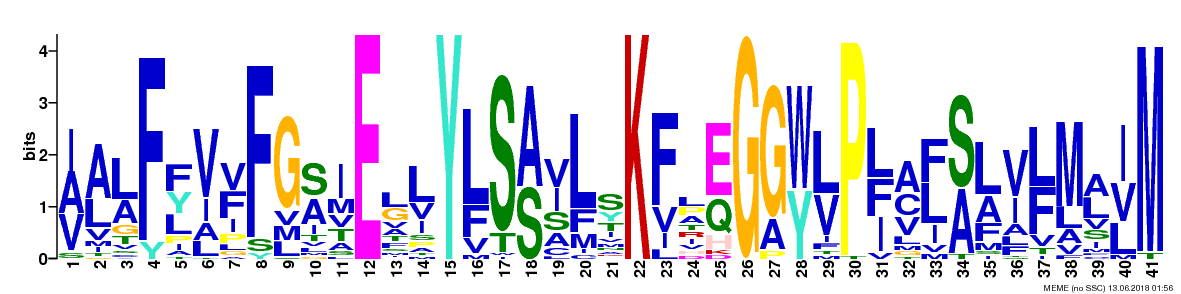 |
|  | IALFFVVFGSIELLYLSAVLSKFLEGGWLPLAFSLVLMAIM |
| Motif11 | 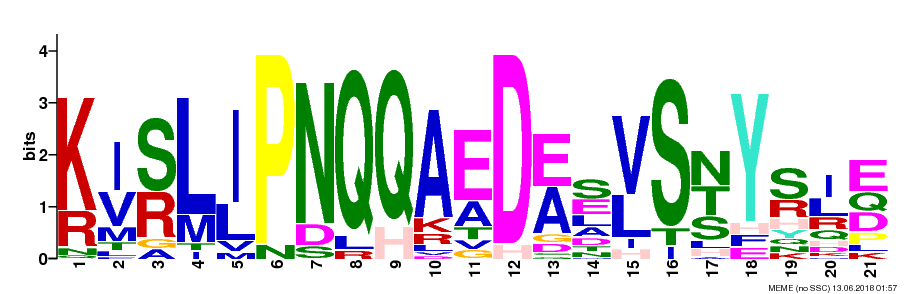 |
|  | KISLIPNQQAEDESVSNYSIE |
| Motif12 | 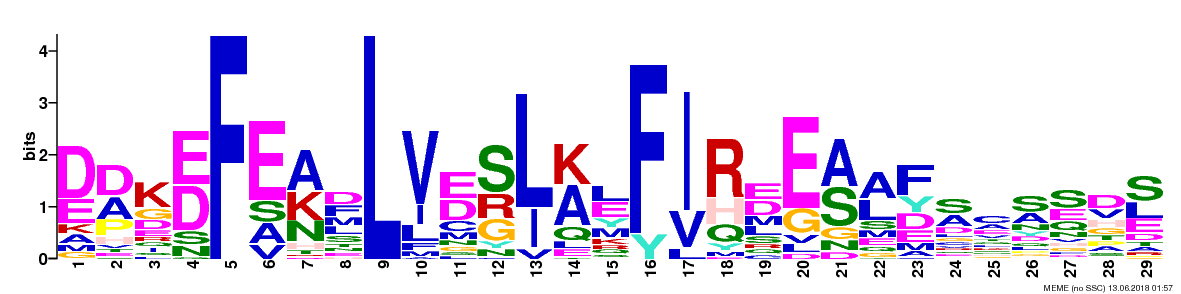 |
|  | DDKEFEADLVESLKEFIREEAAFSCSSDS |
| Motif13 | 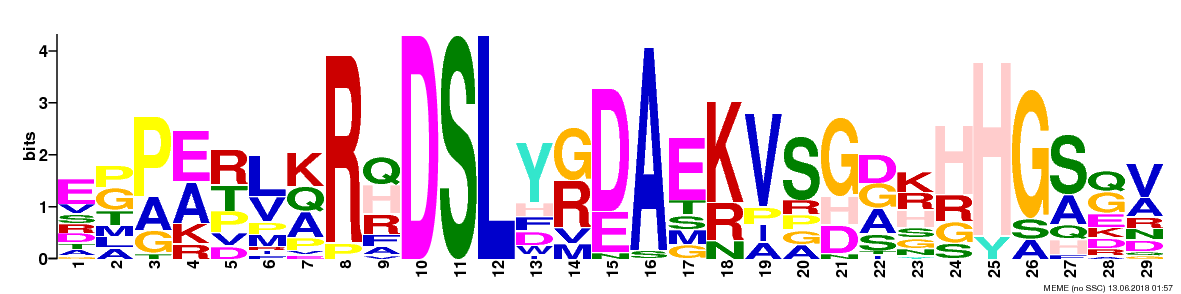 |
|  | EPPERLKRQDSLYGDAEKVSGDKHHGSQV |
| Motif14 | 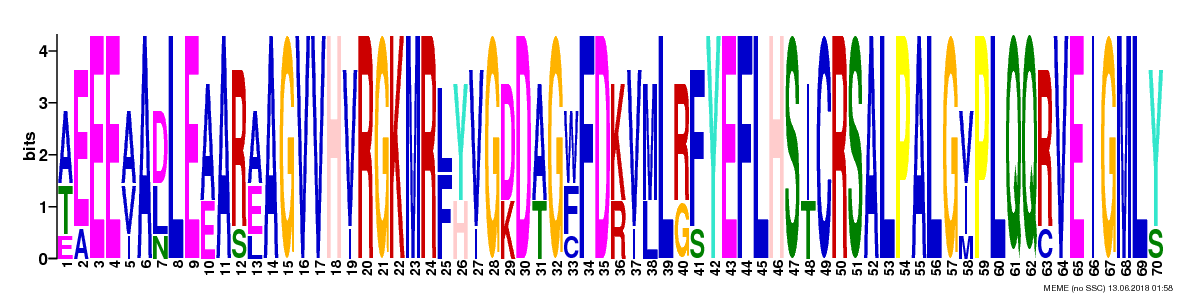 |
|  | AEEEAADLEAARAAGVVHVRGKMRLYVGDDAGWFDKVMLRFYEFLHSICRSALPALGVPLQQRVEIGMLY |
| Motif15 | 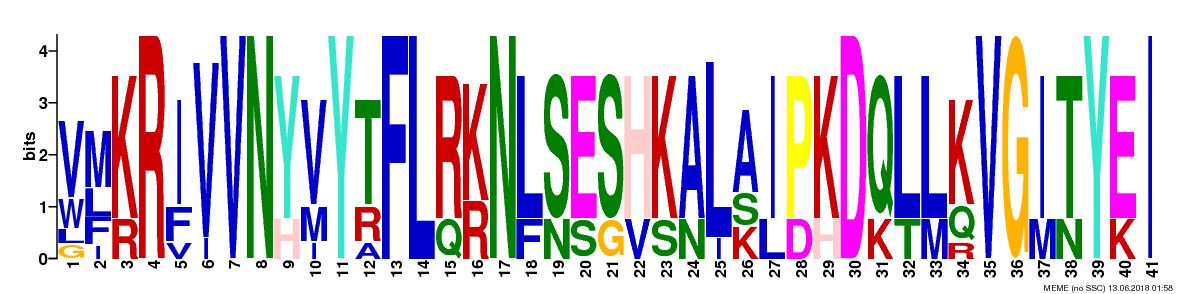 |
|  | VMKRIVVNYVYTFLRKNLSESHKALAIPKDQLLKVGITYEI |
| Motif16 | 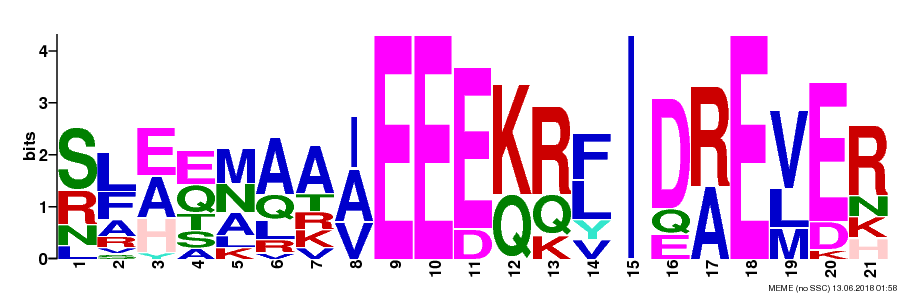   \|  \| \| --- \| |
|  | SLEZMAAIEEEKRFIDREVER |
| Motif17 | 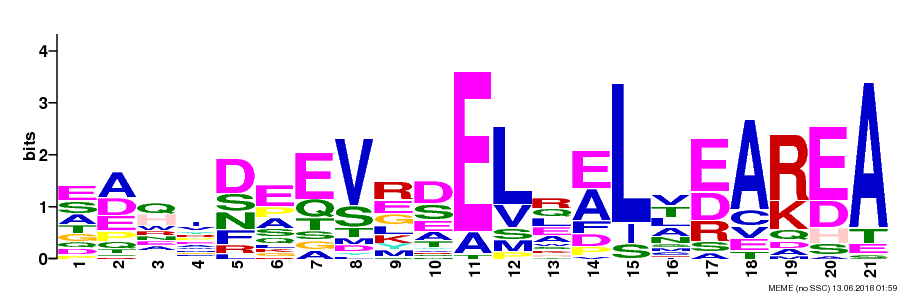 |
|  | EAQIDEEVRDELRELVEAREA |
| Motif18 | 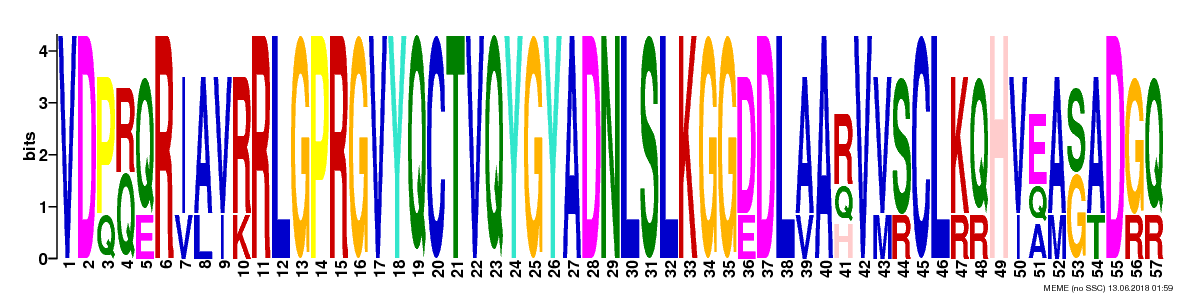 |
|  | VDPRQRIAVRRLGPRGVYQCTVQYGYADNLSLKGGDDLAARVVSCLKQHVEAGADGQ |
| Motif19 | 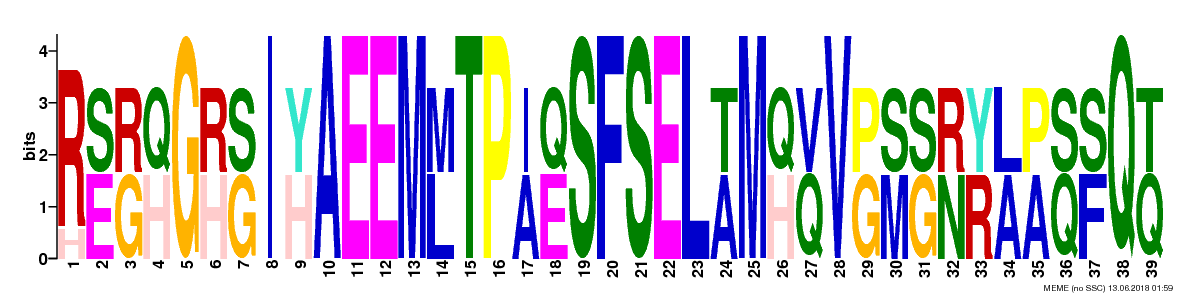 |
|  | REGHGHGIHAEEMLTPIZSFSELAMHVVPSGRYLPQFQT |
| Motif20 | 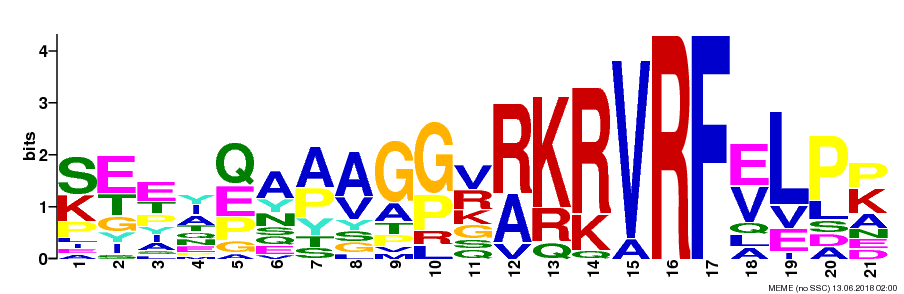 |
|  | SEEAQAAAGGVRKRVRFELPK |
| Motif21 | 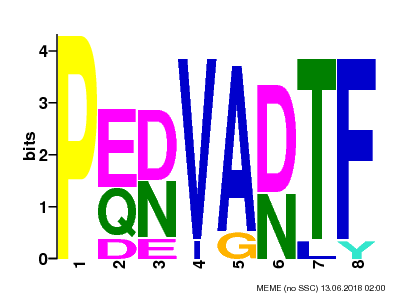 |
|  | PZBVABTF |
| Motif22 | 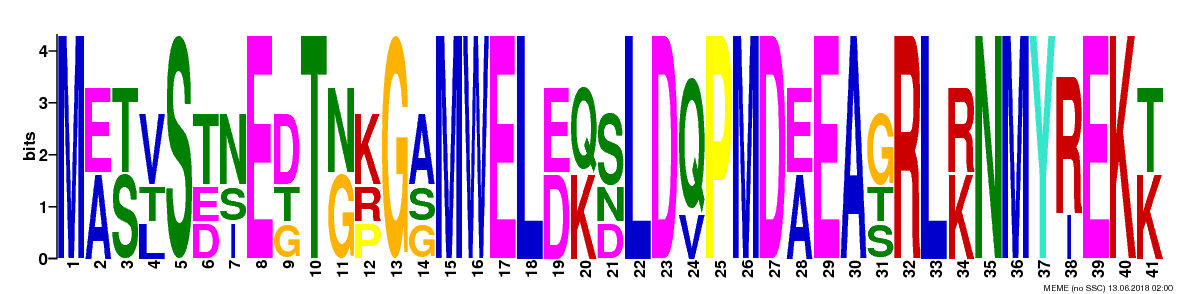 |
|  | METVSTNEDTNKGAMWELDQNLDQPMDEEAGRLKNMYREKK |
| Motif23 | 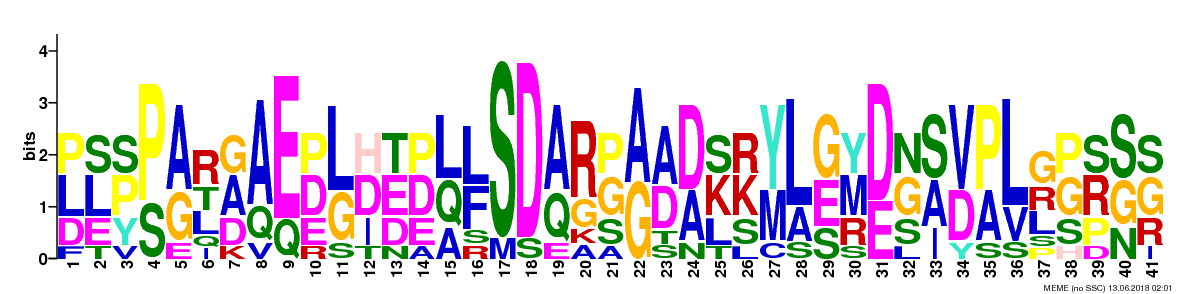 |
|  | PSPPARGAEDLDEDLLSDARPAADKKYLGYDNSVPLGPSSG |
| Motif24 | 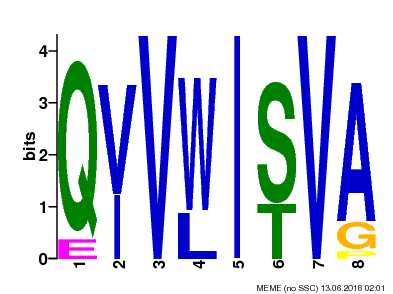 |
|  | QVVWISVA |
| Motif25 | 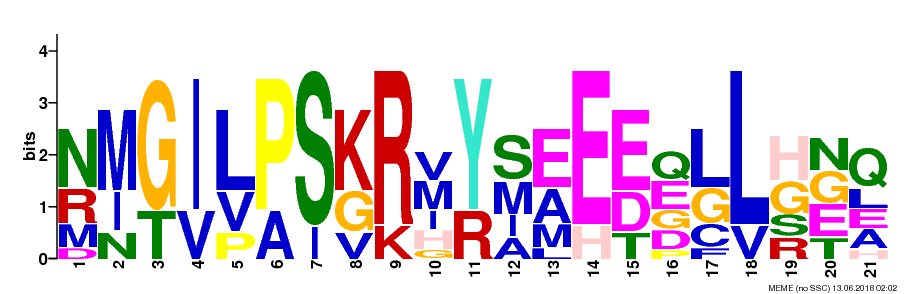 |
|  | NMGILPSKRVYSEEEELLHNQ |
