# Supplementary material for: Genome-Wide Identification and Analysis of HAK/KUP/KT Potassium Transporters Gene Family in Wheat (Triticum aestivum L.)
Source: Int J Mol Sci. 2018 Dec 10;19(12):3969. doi: 10.3390/ijms19123969 (PMC6321448; doi:10.3390/ijms19123969)
Supplement: Supplementary file 1 [file ijms-19-03969-s001.zip › Supplementary files/Table S4.docx]

Table S4. Improved Hogland nutrient solution.

| Solution component | Final concentration (mol/L) |
| --- | --- |
| K_2_SO_4_ | 1.0×10^-3^ |
| MgSO_4_∙7H_2_O | 1.0×10^-3^ |
| CaCl_2_ | 1.5×10^-3^ |
| H_3_BO_3_ | 1.0×10^-6^ |
| (NH_4_)_6_Mo_7_O_24_∙4H_2_O | 5.0×10^-9^ |
| CuSO_4_∙5H_2_O | 5.0×10^-7^ |
| ZnSO_4_∙7H_2_O | 1.0×10^-6^ |
| MnSO_4_∙H_2_O | 1.0×10^-6^ |
| Fe-Na-EDTA | 1.0×10^-4^ |
| Ca(NO_3_)∙4H_2_O | 1.0×10^-3^ |
| NaH_2_PO_4_∙2H_2_O | 2.5×10^-4^ |
| NH_4_NO_3_ | 1.0×10^-3^ |

For the potassium deficiency stress, the final concentration of K_2_SO_4_ was 0.005 mM.

For the salt stress, 200 mM NaCl was added to the above solution.

For the drought stress, 20% PEG 6000 was added to the above solution.
